# A rare case of coexistence of metastasis from head and neck squamous cell carcinoma and tuberculosis within a neck lymph node

**DOI:** 10.1186/s13000-015-0430-x

**Published:** 2015-10-29

**Authors:** Danila Caroppo, Daniela Russo, Francesco Merolla, Gennaro Ilardi, Marialaura Del Basso de Caro, PierPaolo Di Lorenzo, Silvia Varricchio, Massimo Mascolo, Stefania Staibano

**Affiliations:** Department of Advanced Biomedical Sciences, University of Naples “Federico II”, via S. Pansini 5, 80131 Naples, Italy

**Keywords:** Tbc, Lymph node metastasis, OSCC

## Abstract

Coexistence of metastasis from squamous cell carcinoma and tuberculosis within lymph nodes is rare. We report a case of 86 years old woman with a mass in the left laterocervical region. The patient had undergone excision of a poorly differentiated squamous cell carcinoma from the mucosa of the left cheek, a few months before. Histological examination of a mass of few fused lymph nodes, isolated from left laterocervical lymphadenectomy, showed metastatic squamous cell carcinoma with concomitant granulomatous inflammation. A diagnosis of tuberculosis associated with malignancy was posed. The suspect was confirmed by a positive anamnestic finding of a previous tuberculosis infection. The granulomatous reaction may be associated with many types of tumor, and can be found in the draining lymph nodes. The possibility that this reaction is also due to a tuberculosis infection should be kept in mind for elderly oncology patient.

## Background

An association between tuberculosis and cancer has been frequently described. Warthin reported two cases of tuberculosis and carcinoma of mammary glands, in 1899 [1], Kaplan et al. examined the frequency of the coexistence between different cancer types and tuberculosis in a retrospective study [2]. Coexistence, instead, of tuberculosis and metastatic carcinoma in lymph nodes is a rare event; few reports are present in the literature, mainly involving metastases from breast cancer. Herein we report a case of node metastasis from oral squamous cell carcinoma coexisting with an infective granulomatous reaction due to mycobacterium tuberculosis; we discuss clinical, histological and immunohistochemical findings.

## Case presentation

**Fig. 1:**
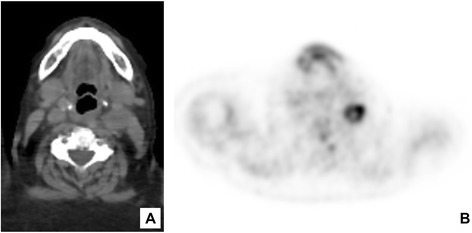
PET-CT: **a** Axial CT image of the maxilla-facial region showing a left retromandibular mass. **b** PET image showing tracer hyperaccumulation at the retromandibular and left parapharyngeal regions

**Fig. 2:**
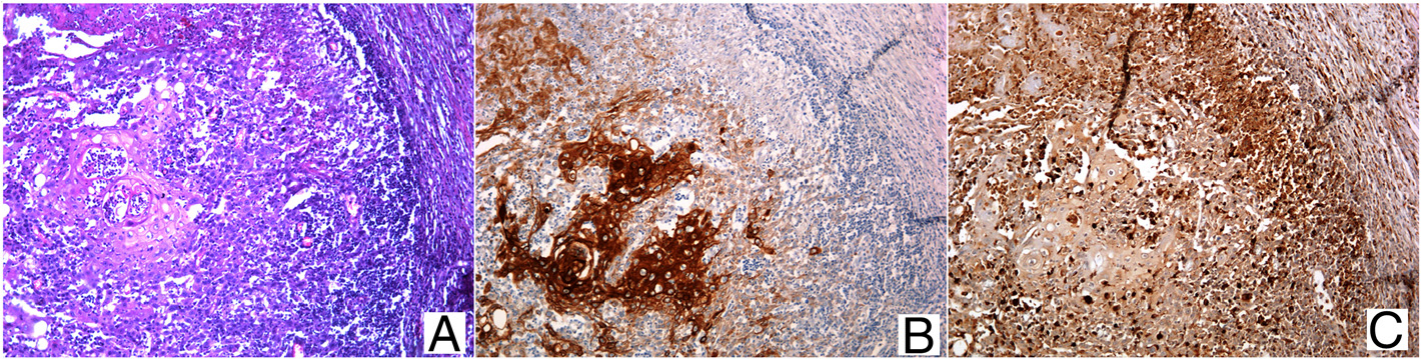
Representative micrograph of the fused lymph nodes: H/E staining showing the granulomatous reaction, with palisading epithelioid histiocytes, and metastatic deposit (**a**); tumor nests are intensely positive to CK pan immunostaining (**b**); CD68 immunostaining confirms the presence of epithelioid histiocytes (**c**). (**a**, **b**, **c** magnification 100×)

A 86-years-old woman, whose smoking habits are not known, noted, in September 2013, the appearance of a small lesion on the left cheek mucosa. She was followed at the department of Oral and Maxillofacial Surgery. The physical examination revealed the presence of an ulcerated lesion, 2 cm of diameter, lightly painful on palpation, with hard/elastic consistence. The histological picture of an incisional biopsy of the lesion, revealed a poorly differentiated, invasive squamous cell carcinoma. The diagnosis was confirmed on the whole excision of the lesion. Three months later, the clinical examination revealed the presence of a node swelling in the left laterocervical region of about 3 cm in diameter, firm, fix and painful on palpation, which showed a rounded morphology at US, ranging in size between 20 and 23 mm of maximum diameter. A PET-CT scan showed tracer hyperaccumulation restricted to the retromandibular and left parapharyngeal regions (SUV 17.4) (Fig. 1a and b). A left laterocervical lymphadenectomy was then performed, and the sample was sent to the institutional Pathology Unit. The surgical specimen was composed of a salivary gland, 12 single lymph nodes and a single mass of few fused lymph nodes (maximum diameter: 3 cm). At microscopic examination, the lymph-node mass showed massive necrosis lined by granulomatous inflammation with palisading epithelioid and Langhans’ giant histiocytes, in close association with metastatic sheets from a squamous cell carcinoma (Fig. 2a). The immunohistochemistry clearly evidenced the tumor nests (pan-cytokeratin positive) and the granulomatous reaction area (CD68 immunoreactive) (Fig. 2b and c). On the basis of morphological and immunohistochemical findings, we posed diagnosis of metastatic squamous cell carcinoma associated with necrotizing granuloma, strongly suggestive of mycobacterial infection/tuberculosis. Requestioning the patient about her previous pathological history, we finally found out that she had an episode of tuberculosis in the 1960s. The patient was redirected to the department of infectious diseases for further investigation; at x-chest rays there was no evidence of pulmonary activity of the disease, as reported for most patients with tuberculosis of the head and neck lymph nodes [3].

Mycobacterium tuberculosis (MTB) infects one third of the world’s population and is the second leading cause of death from an infectious disease after HIV. The association between cancer and tuberculosis infection was already described [1, 2, 4], some authors suggest that the onset of these chronic disease is favored by a state of immunosuppression. Lymphadenitis is the most frequent manifestation of extrapulmonary tuberculosis and neck swelling is the commonest presentation (multiple lymph node widening without constitutional signs). Some authors have reported the presence of tuberculous lymphadenitis in a patient with breast cancer [5] and in a patient with a history of sovraglottic carcinoma [6] who were misdiagnosed as malignant lymph node based on the imaging. Cancer can be associated with a granulomatous response in the draining lymph node [7]; in particular, squamous cell carcinoma can elicit this reaction, being tumor keratins the reaction target [8]. The simultaneous presence of tuberculosis infection and metastasis in the same lymph node is a rare event, described mostly in patients with breast cancer [9–11] and less in patient with a history of head and neck squamous cell carcinoma [8, 12]. Here we describe an unexpected, rare case of node metastasis from oral squamous cell carcinoma coexisting with tuberculosis; to our knowledge, our case represents the first involving the oral cavity as the source of the primary tumor. Wang at al reported two cases of OSCC with node metastasis and TB infection, being the two pathological entities found separately in different lymph-nodes [13].

## Conclusions

In case of node metastasis of head and neck squamous cell carcinoma, the possibility of TBC-lymphadenitis should always be considered even in non-endemic areas, particularly in elderly patients, mainly due to the cancer-induced immune suppression.

## Consent

Written informed consent was obtained from the patient for publication of this case report and accompanying images. A copy of the written consent is available for review by the Editor-in Chief of this journal.

## Abbreviations

HIV: human immunodeficiency virus
MTB: mycobacterium tuberculosis
OSCC: oral squamous cell carcinoma
PET: positron emission tomography
Tbc: tubercolosis
US: ultra sounds
